# How Native Prosody Affects Pitch Processing during Word Learning in Limburgian and Dutch Toddlers and Adults

**DOI:** 10.3389/fpsyg.2017.01652

**Published:** 2017-09-22

**Authors:** Stefanie Ramachers, Susanne Brouwer, Paula Fikkert

**Affiliations:** ^1^Department of German Language and Culture, Centre for Language Studies, Radboud University Nijmegen, Netherlands; ^2^Department of Dutch Language and Culture, Centre for Language Studies, Radboud University Nijmegen, Netherlands

**Keywords:** lexical tone, word learning, word recognition, preferential looking, bidialectalism, Limburgian, mispronunciations

## Abstract

In this study, Limburgian and Dutch 2.5- to 4-year-olds and adults took part in a word learning experiment. Following the procedure employed by Quam and Swingley (2010) and Singh et al. (2014), participants learned two novel word-object mappings. After training, word recognition was tested in correct pronunciation (CP) trials and mispronunciation (MP) trials featuring a pitch change. Since Limburgian is considered a restricted tone language, we expected that the pitch change would hinder word recognition in Limburgian, but not in non-tonal Dutch listeners. Contrary to our expectations, both Limburgian and Dutch children appeared to be sensitive to pitch changes in newly learned words, indicated by a significant decrease in target fixation in MP trials compared to CP trials. Limburgian and Dutch adults showed very strong naming effects in both trial types. The results are discussed against the background of the influence of the native prosodic system.

## Introduction

Acquiring the sound structure of a language entails finding out which phonetic contrasts are meaningful in the native language (L1) and storing them as part of a word’s lexical representation. Children need to learn to assign appropriate interpretations to many different sorts of phonetic variation, and separate variation that is lexically meaningful (i.e., phonemic variation) from variation that is not (e.g., speaker variation). Many studies have looked into the developmental perception of speech sound contrasts in the first year of life and into the way they are processed during word learning and recognition at later ages (e.g., Jusczyck, 1997; Stager and Werker, 1997; Swingley and Aslin, 2000; Kuhl, 2004; White and Morgan, 2008). This research has focused mainly on segmental contrasts, whereas approximately 60–70% of the world’s languages employ pitch differences to distinguish words in addition to vocalic and consonantal contrasts (Yip, 2002). The aim of the present study is to add to the field of lexical tone acquisition by investigating the role of pitch contrasts during novel word learning. This is examined in child and adult speakers of Limburgian dialects of Dutch. Limburgian^1^ is a restricted tone language yielding an intriguing interaction between lexical and intonational tones. Limburgian participants’ performance in a word learning experiment is compared to a control group of monolingual child and adult speakers of Dutch.

Pitch variation is meaningful in all languages of the world (Yip, 2002; Gussenhoven, 2004; Singh and Fu, 2016). Tone languages such as Mandarin Chinese use pitch to distinguish words, similar to what phonemes do at the segmental level. Some tone languages make very extensive use of lexical pitch. Mandarin Chinese specifies every mora for tone, ignoring toneless neutral syllables (Duanmu, 2000). Other tone languages are more restricted in their use of lexical pitch. These languages, for example Tokyo Japanese, have been referred to as either PITCH-ACCENT LANGUAGES or RESTRICTED TONE LANGUAGES (Voorhoeve, 1973; Hyman, 2009). Whether there is a clear-cut distinction between tone languages and restricted tone languages is heavily debated. What they have in common is that pitch, be it to a greater or lesser extent, is necessary for determining the meaning of a word. Following Hyman’s (2001, 2009) definition, we take the term ‘tone language’ to refer to languages that use pitch to distinguish between words.

Importantly, in non-tone languages like Dutch and English, pitch is not used to distinguish between words – except in a few very rare minimal pairs that differ in word stress (e.g., Dutch *VOORkomen* ‘appear’ vs. *voorKOMEN* ‘prevent’), in which case pitch is only one of several correlated cues to stress. The fact that pitch is not lexically distinctive in non-tone languages might prevent speakers of these languages from distinguishing monosyllables that differ in pitch only (Schaefer and Darcy, 2014) and from encoding pitch information when building novel lexical representations (Braun et al., 2014).

Despite the abovementioned functional differences, non-tone language listeners often show sensitivity to non-native lexical tones (e.g., Hallé et al., 2004; So and Best, 2010, 2014; Liu and Kager, 2014; Ramachers et al., 2017). This sensitivity is mostly shown in perceptual tasks without lexical involvement (i.e., discrimination tasks; e.g., Broselow et al., 1987; So and Best, 2008, 2010, 2014; Liu and Kager, 2014; Schaefer and Darcy, 2014; Ramachers et al., 2017). Several factors have been put forward recently to account for these findings, the most important one being the role of prosody in the L1.

The PERCEPTUAL ASSIMILATION MODEL FOR SUPRASEGMENTALS (PAM-S; So and Best, 2014) states that non-native pitch contrasts tend to be perceived according to their degree of similarity to native pitch patterns. Indeed, a number of studies on the perception of non-native pitch patterns have shown that prosodic experience from listeners’ L1 guides their perception of non-native pitch patterns (e.g., Broselow et al., 1987; So and Best, 2008, 2010, 2014). For example, English listeners presumably discriminate Mandarin tone 4 (falling) due to assimilation to their statement intonation category (e.g., Broselow et al., 1987; So and Best, 2008), and Dutch listeners in Braun and Johnson (2011) probably perceived utterance-final Mandarin tone 2 (rising) as Dutch question intonation. Following these observations, the question thus no longer is whether non-tone language listeners discriminate lexical tones, but whether they interpret them as lexically relevant.

When acquiring a lexicon, tone language learners need to learn to ascribe lexical relevance to pitch changes and encode tone lexically. Conversely, non-tone language learners have to learn to disregard pitch changes that occur within words, despite the fact that they might still discriminate these pitch changes at lower levels of processing (e.g., in a purely perceptual task).

### Integration of Pitch into Lexical Representations

Recent work suggests that child and adult speakers of tone languages behave differently from non-tone language speakers in exploiting contrastive pitch contours when learning words. Tone language speakers attend to pitch information and exploit it during lexical access, whereas non-tone languages speakers do not, or at least to a lesser extent (e.g., Quam and Swingley, 2010; Braun et al., 2014; Singh et al., 2014; Hay et al., 2015). These previous studies primarily discussed the lexical integration of pitch by non-tone language listeners. Few of them looked at the interpretation of (non-)native pitch by tone language listeners, and if so, they focused on typically studied tone languages like Mandarin Chinese. However, within the family of tone languages, large differences exist.

First, tone languages differ with respect to the functional load of tone, which depends on the tonal inventory (i.e., the number of tones, and, related to that, their information value), the distributional restrictions of tones (i.e., can they appear on any syllable?), the importance of tones for lexical disambiguation (i.e., how many minimal pairs are there in the language?), and the extent to which f0 is the only cue to the tonal distinction (i.e., do duration or voice quality play a role?) (e.g., Pierrehumbert and Beckman, 1988; Kristoffersen, 2000; Wang et al., 2004; Tong et al., 2008; Wu et al., 2012). The smaller the inventory, the larger the amount of distributional restrictions and the smaller the number of tonal minimal pairs, the more restricted a tone system is (Voorhoeve, 1973). The functional load of lexical pitch patterns in the L1 has been assumed to influence sensitivity to word-level pitch in speakers of these languages (e.g., Wang et al., 2004; Wu et al., 2012; Schaefer and Darcy, 2014; Goss, 2015).

A second difference within the family of tone languages lies in the complexity of their intonation systems. Typically, tone languages do not have complex intonation systems (e.g., Gussenhoven and van der Vliet, 1999) and, as a consequence, the pronunciation of a word with a certain lexical tone is rather stable across different contexts. In Standard Chinese, for example, different intonations only cause changes in pitch height, not in pitch contours (Wu, 2000). However, some more restricted tone systems, like Norwegian, Swedish, and Limburgian, do show complex intonation systems. In these languages, intonation tones interact with lexical tones, causing variation in surface realizations (i.e., contours) of a lexical tone (e.g., Gussenhoven, 2000a; Riad, 2013). It has been suggested that surface variability in the contours of lexical tones can delay the acquisition of lexical tone assignment (Demuth, 1995; Ota, 2003).

In the present study, we investigated lexical encoding of tone in Limburgian. By studying a language with a low functional load for a binary tone contrast embedded in a complex intonation system, this study widens our understanding of the influence of the functional load of tone and tonal surface variability on the acquisition and processing of a lexical tone system. By comparing Limburgians to a control group of non-tonal Dutch peers, we also address the influence that cross-linguistic differences in the functionality of pitch have on pitch processing. Before elaborating on Limburgian, we first review the existing literature that typically studied the lexical integration of pitch in non-tone language speakers and/or in tone languages with a high functional load for tone.

Quam and Swingley (2010) tested recognition of newly learned words carrying a tone in a bimodal preferential looking experiment adopting a mispronunciation paradigm. The idea behind mispronunciation paradigms is that successful detection of form-meaning mismatches requires the prior establishment of novel representations that include the tonal or segmental specification of interest. If the lexical representation of the newly acquired word is impoverished or incomplete with respect to for example its tonal specification, word recognition will not be hindered by tonal variability in the input signal.

In their study, English 30-month-old toddlers and adults were taught a novel pseudo-word as a label for a new toy. Subsequently, the target was either correctly pronounced (CP), i.e., with the trained tone, or mispronounced (MP), i.e., with a change in tone or a change in vowel. Quam and Swingley (2010) showed that both children and adults interpreted the changes in accordance with their native phonology. Word recognition was hindered by a vowel change, but not by a change in pitch. At least by 30 months of age, English children have thus learned to disregard pitch at the level of words.

In a paradigm similar to that of Quam and Swingley (2010), Singh et al. (2014) showed that, at 18 months, mono- and bilingual English learners were equally sensitive to tonal and vowel MPs, but at 24 months they no longer treated pitch as lexically contrastive, in accordance with their native phonology and in line with Quam and Swingley (2010). Mandarin-English bilinguals^2^ who were dominant in Mandarin were sensitive to both vowel and tonal MPs at both ages. The authors suggest that, at 18 months, toddlers may over-assign weight to post-lexical pitch information due to its high attentional appeal and by virtue of having observed its linguistic significance, either at the post-lexical or at the paralinguistic level.

Similar findings come from a series of experiments by Hay et al. (2015). In an associative word learning task using the two-object switch procedure (Stager and Werker, 1997), 14-month-old but not 17- and 19-month-old learners of English interpreted pitch differences as properties of words. According to Hay et al. (2015, p. 10), between 14 and 17–19 months, children go through a phase of “interpretive narrowing.” With growing linguistic experience, they become more specific about what forms of words should be treated as lexically contrastive. Nevertheless, 17- and 19-month-olds continued to be sensitive to the difference between falling and rising pitch contours in a discrimination task that did not involve label-object mappings. To sum up, the studies above show that there is a shift in English children’s interpretation of the lexical relevance of pitch patterns in the course of the second year of life.

A study that compared the ability to store lexical tones (in this case Mandarin tones) among *adult* speakers of languages differing in their lexical and post-lexical use of prosody is reported in Braun et al. (2014). The languages under investigation (German, Japanese, French, and Mandarin) differed with respect to the *lexical* status of word-level prosody as well as the complexity of the *post-lexical* pitch system (i.e., the number of utterance-level contrasts). German, a stress language, makes use of word-level prosody. Moreover, it has a relatively rich intonational system. French does not assign word stress to lexical items and would appear to have less pitch variability at the utterance-level. Japanese has word-level prosody in the form of pitch-accents. However, as in French, utterance-level pitch variability is more restricted. Speakers of Mandarin, Japanese, German, and French had previously shown sensitivity to Chinese tones in purely perceptual tasks.

The aim in Braun et al. (2014) was to see if the ability to lexically encode pitch in a word learning paradigm depended on experience with lexical or post-lexical prosody. Participants’ recognition of newly learned words was tested in tonal and segmental mismatch conditions. As hypothesized, performance was modulated by the different prosodic structures of the participants’ L1. The Mandarin group outperformed all the other groups. More surprisingly, German participants significantly outperformed Japanese and French listeners. Japanese and French listeners did not differ significantly from each other. The authors argue that the number of L1 *utterance-level* pitch contrasts, rather than the availability of *word-level* pitch contrasts, are beneficial for building long-term representations of lexical tone. However, German participants might have benefited both from their experience with f0 as a cue to word stress and as a cue to post-lexical intonation. Importantly, the fact that f0 is hardly used to signal *lexical* distinctiveness in German obviously does not prevent them from perceiving and lexically encoding pitch information.

Much less is known about the lexical integration of pitch by speakers of more restricted tone languages like Limburgian. The next section provides more information on the lexical tone system in Limburgian.

### The Limburgian Dialects of Dutch

**FIGURE 1:**
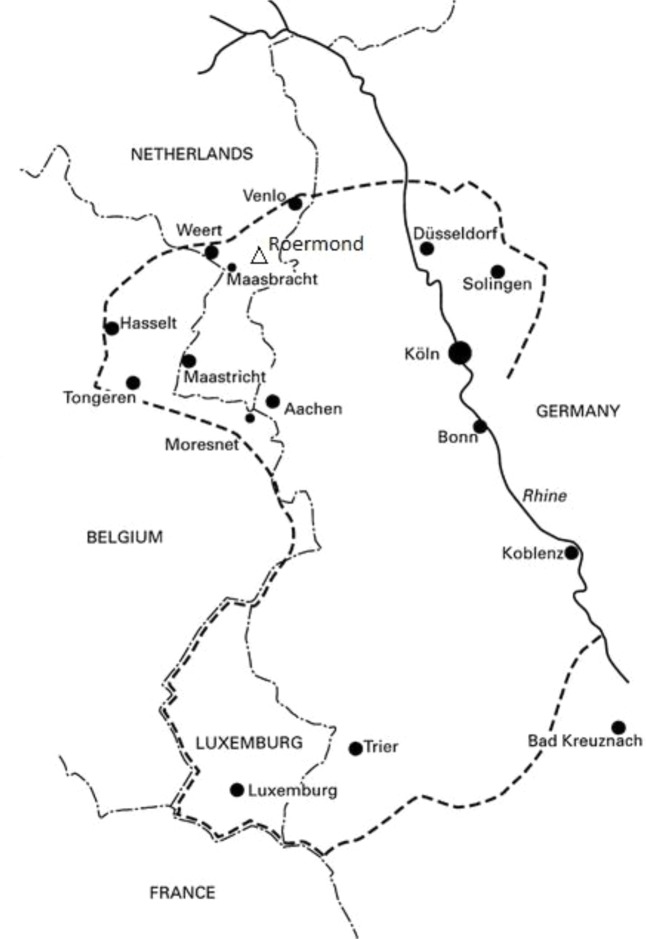
Geographical distribution of the lexical tone contrast in the Cologne-Trier area. Adapted from Gussenhoven and Bruce (1999).

The Limburgian dialects of Dutch belong to the Central Franconian dialect-continuum which covers the provinces of Limburg in the Netherlands and Belgium as well as the north of the German Rhineland-Palatinate and the southwest of North-Rhine Westphalia (Gussenhoven, 2000a; Fournier, 2008; see **Figure 1**).

The Dutch province of Limburg has about 1.1 million inhabitants^3^, 75% of which speak a Limburgian dialect (Driessen, 2006). Limburgian is a regional linguistic variety of Standard Dutch, the official language used in formal and institutional settings. Differences exist at the phonological, morphosyntactic, and lexical level, but still, mutual intelligibility is fairly high (Van Bezooijen and Van den Berg, 1999) due to the existence of many cognates. The probably most striking difference between Limburgian and Dutch is the fact that many Limburgian dialects have lexical tone.^4^ Pitch is used in both languages as a cue to word stress and in post-lexical intonation (e.g., Gussenhoven, 1988; Gussenhoven and van der Vliet, 1999).

In this study, the focus is on the dialect of Roermond. The choice to focus on one particular dialect instead of on Limburgian as a whole stems from the fact that Limburgian is not a homogeneous linguistic variety. Limburgian is to be understood as an umbrella term for many different dialects. Comparable to the pitch-accents in different varieties of Japanese, Norwegian, and Swedish (Wetterlin, 2007; Tamaoka et al., 2014), the Limburgian tones may have different phonetic realizations across dialects, be embedded in different intonational systems or may be absent altogether (e.g., Gussenhoven, 2000a; Gussenhoven and Peters, 2008). The choice for the dialect of Roermond is partly motivated by the existence of a series of tone perception and production studies with adult speakers of Roermond Dutch (Fournier et al., 2006; Fournier, 2008; Fournier et al., 2010). Moreover, its vocabulary and (tonal) grammar are well documented (e.g., Kats, 1939, 1985; Gussenhoven, 2000b).

**FIGURE 2:**
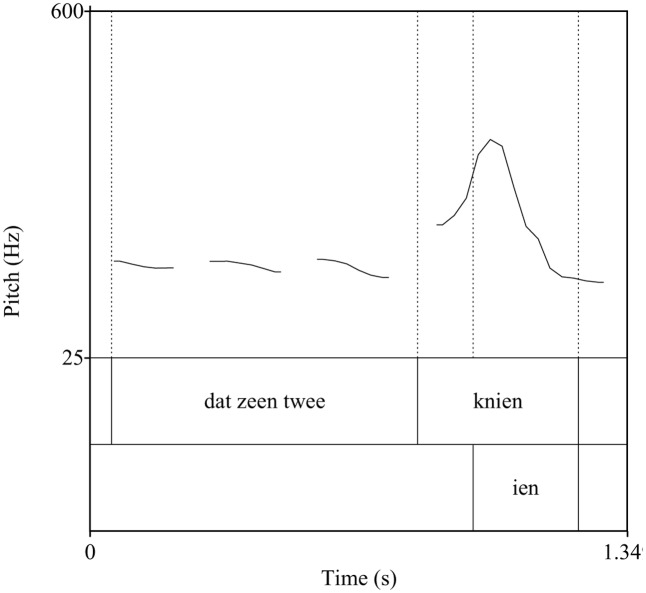
F0 contour of the Limburgian sentence *dat zeen twee KNIEN* ‘those are two rabbits.’ The rhyme of the target word carries accent 1.

**FIGURE 3:**
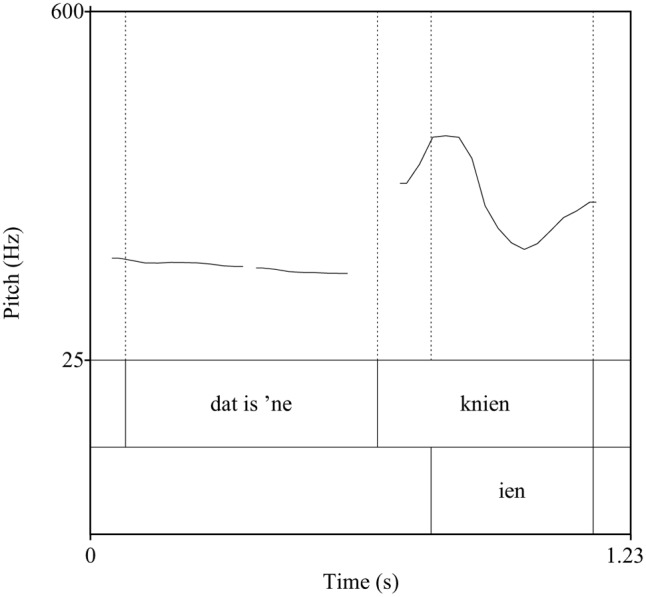
F0 contour of the Limburgian sentence *dat is ‘ne KNIEN* ‘that is a rabbit.’ The rhyme of the target word carries accent 2.

In Roermond Dutch, *haas* [ha:s] with falling pitch (accent 1) means ‘hare,’ whereas *haas* with falling-rising pitch (accent 2) means ‘glove.’ In a small number of frequent nouns, pitch also serves a grammatical function with accent 1 systematically indicating plurality (see **Figures 2**, **3**). In the Roermond dialect, the primary acoustic cue to the tone contrast is f0.

Lexical tone in Limburgian^5^ has a lower functional load than tone in many Chinese dialects. There are few minimal pairs (approximately 80; Fournier, 2008), and there is only a two-way contrast. Gussenhoven and Peters (2008, p. 88) assume that “the word accent contrast (…) amounts to a contrast between the absence of lexical tone (Accent 1) and its presence (Accent 2).” Moreover, the contrast can only be realized on syllables with main stress, meaning that an unbound multisyllabic morpheme can only carry one accent. For this reason, Limburgian is comparable to for example Japanese (Kubozono, 1993; Tamaoka et al., 2014), Swedish (Gussenhoven, 2004; Riad, 2013), and Norwegian (Kristoffersen, 2000; Wetterlin, 2007; Steien and Van Dommelen, 2016). With respect to the domain of realization of lexical tone, Limburgian is more akin to tone languages such as Mandarin (Burnham et al., 2014), as the pitch contrast is realized within a single syllable.

Apart from the relatively small number of minimal pairs, any primary stressed bimoraic syllable is pronounced either with accent 1 or with accent 2 (Gussenhoven, 2000b). For example, in Roermond Limburgian, *boum* [bɔʊm] (‘tree’) carries accent 2, whereas *sjaop* [ʃɔ:p] (‘sheep’) carries accent 1. Pronouncing any of these words with the wrong accent would turn them into a non-existing word. Pitch is thus assumed to be part of a word’s mental representation.

By studying Limburgian speakers’ sensitivity to pitch changes, we could shed more light onto the lexical representations of accent 1 and accent 2. The FEATURALLY UNDERSPECIFIED LEXICON MODEL (Lahiri and Reetz, 2002) can be used to formulate predictions on this matter. If the lexical representation of a word is incomplete with respect to its tonal specification, tonal features present in the input signal cannot mismatch with an underspecified (i.e., empty) slot in the lexicon. In this case, word recognition cannot be hindered by tonal variability in the input. If it is indeed the case that accent 2 is the underlyingly specified accent, Limburgians would be sensitive to mispronunciations of accent 2 (leading to a *mismatch*), but not or to a lesser extent to mispronunciations of accent 1 (leading to a *no-mismatch*).

As in any other language, pitch in Limburgian also serves post-lexical functions. Limburgian dialects have complex intonation systems (Gussenhoven and van der Vliet, 1999). As a result, the pitch contours of the accents vary as a function of information status, sentence type, and position in the utterance. Surface variation due to tone-intonation interactions can also be observed in Swedish (Bruce, 1977; Riad, 2013), but to a lesser extent than in Limburgian (Gussenhoven, 2004). It has been suggested that the reliability of the mapping between underlying tones and their surface realizations has a large impact on the acquisition of a lexical tone system (Demuth, 1995; Ota, 2003). In addition, Rost and McMurray (2010) have shown that allophonic variability, unlike variability like speaker differences, can be problematic for creating phonologically specific representations of new words. Children might have a hard time distinguishing allophonic from phonemic variation, not knowing what to add to their lexical representations, leading to initially/temporarily under- or over-specified representations. Limburgian listeners are confronted with a considerable amount of allophonic (or *allotonic*) variation in lexical tone contours. Furthermore, this variation cannot be ignored since it does signal meaningful information at the post-lexical level. In light of this variation, it could be a challenge to recover the underlying tone system for young learners of Limburgian.

Yet another source of variation in Limburgians’ input is due to the fact that most Limburgians also speak Dutch and are considered bidialectal (Cornips, 2014). Hardly any studies on the mapping of sounds to meaning focused on children acquiring two languages, let alone on children acquiring multiple dialects or regional varieties of the same language (for a review, see Fennell et al., 2016). Extant studies have shown that learning novel minimal pair words in both mono- and bilinguals is favored when children listen to a speaker that sounds like people from their environment (e.g., Mattock et al., 2010; Fennell and Byers-Heinlein, 2014). In word recognition studies with known words, the use of cognates can hinder the detection of mispronunciations, at least in close-language bilinguals (e.g., Ramon-Casas and Bosch, 2010). As a consequence of the highly variable input Limburgians are exposed to (Durrant et al., 2015), the higher probability of hearing accented speech (e.g., Bosch and Ramon-Casas, 2011) and the large amount of lexical overlap in the input (e.g., Sebastián-Gallés and Bosch, 2009), Limburgian children might exhibit a more lenient treatment of mispronunciations.

### Aims of the Present Study

In this study, we ask whether pitch plays a role in novel word recognition for children acquiring Roermond Limburgian in comparison to a control group of children acquiring Dutch. We aimed to answer two questions. First, do children acquiring Roermond Limburgian encode pitch information as part of their lexical entries when learning novel words? And secondly, do they behave differently from Dutch age-matched peers in this respect? To see whether their interpretation of pitch is adult-like or not yet fully developed, we also tested Limburgian and Dutch adults. Limburgian and Dutch 2.5- to 4-year-olds (Experiment 1) as well as adults (Experiment 2) participated in a bimodal preferential looking experiment (Golinkoff et al., 1987). Following the procedure employed by Quam and Swingley (2010) and Singh et al. (2014), participants learned two novel word-object mappings. After training, word recognition was tested in correct pronunciation (CP) trials and mispronunciation (MP) trials featuring a pitch change.

In light of previous findings (Singh et al., 2014, 2015), we expected Limburgians to be sensitive to MPs involving pitch. However, a change in pitch might only hinder word recognition to a minor extent in Limburgian due to the relatively restricted nature of the Limburgian tonal system. Another characteristic of the Limburgian speakers’ input that could lead to (temporarily) weaker MP effects is the large amount of surface variation in the contours of the Limburgian tones, phonetic variation due to their exposure to multiple regional variants of a language (Durrant et al., 2015), and possibly also the fair amount of Dutch cognates without a tonal specification (but see Van der Feest and Johnson, 2016).

As for our Dutch participants, Ramachers et al. (2017) have shown that Dutch 6- to 12-month-old infants reliably discriminate the Limburgian tones in a discrimination task (see also Liu and Kager, 2014; Chen and Kager, 2016). Here we ask whether Dutch participants still attend to pitch in a higher-level task that requires lexical encoding of pitch. Based on previous research with non-tone language speakers (e.g., Quam and Swingley, 2010; Singh et al., 2014; Hay et al., 2015), we expected that changes in pitch would not hinder Dutch subjects’ recognition of newly learned words.

However, adult speakers of German showed sensitivity to word-level pitch differences despite the fact that German has no lexical tone (Braun et al., 2014). Also, de Bree et al. (2008) showed that Dutch 36-month-olds were sensitive to miss-stressing. The fact that 3-year-old Dutch children appear to be sensitive to word-level suprasegmental properties might also facilitate their encoding of other word-level prosodic features, like lexical tone.

For the adults, in principle the same expectations hold. However, due to accumulated linguistic experience, Limburgian adults might have learned not to rely on pitch alone during online language comprehension. We expected Limburgian adults to notice a change in tone, but it is an open question how strongly it will hinder word recognition. Dutch adults might also still show sensitivity to pitch differences by virtue of their accumulated linguistic experience with post-lexical intonation and word stress (but see Quam and Swingley, 2010).

## Experiment 1

### Materials and Methods

#### Participants

A total number of 41 Limburgian toddlers were recruited via health care institutions and daycare centers in the city of Roermond in the Dutch province of Limburg. Twenty-three children with a mean age of 40.9 months (*SD* = 5.9 months; range = 31–49 months; 6 boys) were included in the analysis. An additional 18 toddlers were tested but excluded from analysis because they failed to contribute sufficient data. For a detailed description of trial, block and participant exclusion criteria we refer to the section “Data Pre-processing and Analysis” and **Table A1** in the **Appendix**.

Children in Limburg are often exposed to quite heterogeneous linguistic input. As a result, it is difficult to find toddlers who have only been exposed to one particular dialect, in our case Roermond Limburgian. Children from the municipality of Roermond who were exposed to any East-Limburgian dialect (Bakker and van Hout, 2012), spoken by at least one parent or caregiver, were allowed to participate. The realization of the word prosodic contrast within the East-Limburgian dialect region does not show much variation (Heijmans, 2003). Based on parental report (missing *N* = 1), using an adapted version of the PaBiQ (COST Action IS0804, 2011)^6^ administered during a telephone interview, the language input provided at home to 22 of the Limburgian children was as follows: (a) both parents speak a different East-Limburgian dialect (*N* = 9), (b) one parent speaks an East-Limburgian dialect, the other Standard Dutch (*N* = 8), (c) both parents speak the same East-Limburgian dialect (*N* = 3), and (d) one parent speaks an East-Limburgian dialect, the other a dialect from another Limburgian dialect region (*N* = 2). All children were reported to understand both Limburgian and Dutch. Moreover, 19 out of 22 children were reported to speak Limburgian, and all participants were reported to speak Dutch. All Limburgian toddlers thus picked up on Dutch, even if they were not addressed in it by (one of) their parents, but for example by friends or at daycare. All toddlers could thus be considered bidialectals. For language use in the home (input quantity) parents were asked a series of questions with rating scale responses about the languages used by each household member to the child. From this, a proportion of language use in the home was derived. The questionnaire also contained a language richness measure (input quality), as defined by the extent to which children were exposed to story-telling, either as read from books or produced spontaneously, the expression of feelings, educational games (e.g., counting and spelling), labeling new objects, and media (e.g., television, PC, and tablet). Eighteen out of twenty-two children had higher input quantity scores in Limburgian than in Dutch. Seventeen out of twenty-two children had higher or equal input quality scores in Limburgian than in Dutch. See **Table A2** in the **Appendix** for more details.

A total number of 40 Dutch toddlers were recruited from the subject pool of the Baby Research Center of Radboud University, Nijmegen, Netherlands. All infants grew up in monolingual Dutch-speaking families. Thirty-five toddlers with a mean age of 36.8 months (*SD* = 1.8 months; range = 34–40 months; 13 boys) were included in the analysis. An additional five participants were excluded from the analysis for not contributing enough data (*N* = 4) and because one pair of children were twins (*N* = 1; the child contributing the least number of trials was excluded).

To make sure that none of the Dutch toddlers had substantial experience with a Limburgian dialect or any other tone language, their parents were asked questions related to the linguistic input of their child during an intake phone call. A child was regarded to have substantial experience with a tone language and thus not suitable for participation if: (a) one of the parents or primary caregivers was a native speaker of a tone language, (b) the child had weekly contact with a native tone language speaker.

None of the participants had known developmental disorders or delays and none of them had substantial exposure to a language other than Limburgian or Dutch. Ethical approval for the study was obtained from the Ethiek Commissie Faculteit der Sociale Wetenschappen (ECSW) at Radboud University in Nijmegen, Netherlands. Caregivers signed an informed consent and received a picture book or a small monetary compensation for their participation.

#### Apparatus

Limburgian children were tested in a dimly lit office using a portable lab set-up in a daycare center in Roermond. They sat in front of a 24-inch LCD screen (Philips 249C4QHSB) and were recorded via a digital video camera (Sony HC40) mounted on a tripod below the table. Behind the monitor were two speakers (Logitech Z130). The video camera broadcast the recording to a 13-inch Apple MacBook Air. Recordings were made with the video software Vidi (version 0.4.7). The experiment was presented using the LOOK software (Meints and Woodford, 2008), run on a laptop (HP EliteBook Folio 9470m). During testing, experimenter and caregiver listened to masking music through noise-canceling headphones (Sennheiser HME 110).

Dutch children were tested in a dimly lit room in the Baby Research Center at Radboud University, Nijmegen, Netherlands. The experiment was run in a test booth (size: 128 cm × 177 cm), which is partly closed by black wooden partitions, left and right from the 47-inch television screen (LG 47LK530 ZC). A digital video camera (Sony Handycam DCR_HC85E PAL) was placed 30 cm below the screen, hidden by a black curtain with an opening for the lens. The video camera provided a broadcast of the infant’s behavior to a monitor behind the TV. Recordings for offline coding were made using Virtual Dub (Version 1.9.11). The experiment was controlled using the LOOK software (Meints and Woodford, 2008). Experimenter and caregiver wore noise-canceling headphones (Sennheiser HMEC 300) that played masking music.

#### Procedure

**FIGURE 4:**
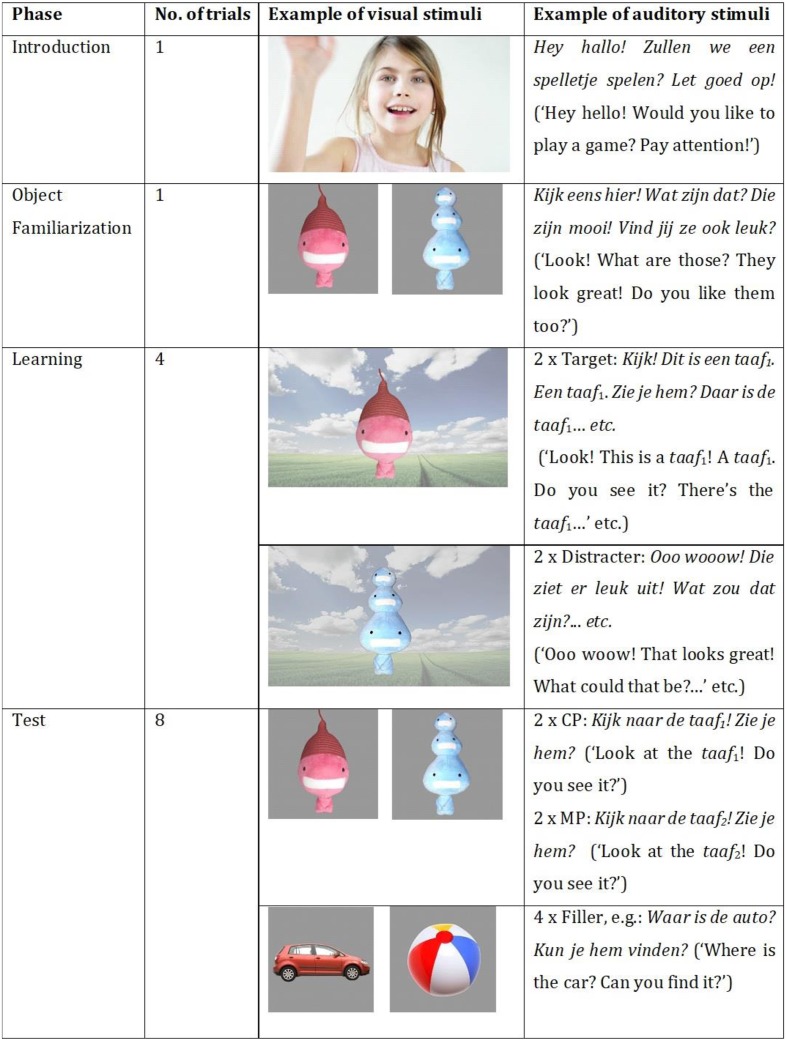
Visual overview of an experimental block.

The procedure employed was the intermodal preferential looking paradigm (Golinkoff et al., 1987). The experiment lasted approximately 10 min and consisted of two blocks, separated by a 1-min break. In each block, children would learn one novel word-object mapping and subsequently it was tested how they reacted to a pitch change in the newly learned word. Each child thus learned two new words, one with accent 1 and one with accent 2. Half of the participants learned the accent 1 word first and half learned the accent 2 word first. Each block featured a different pair of objects. A visual overview of a block is presented in **Figure 4**.

A block started with an encouraging introduction phase inviting the participant to play a game. In the following object familiarization phase, the child was familiarized with two novel toy objects appearing simultaneously at the far left and far right side of the screen. The objects were presented for 9 s. The child heard (in Limburgian or in Dutch): “Look! What are those? They look great! Do you like them too?” One of these objects (the target) would be labeled in the subsequent learning phase. The other one (the distracter) would remain nameless. Target side during object familiarization was counterbalanced across blocks. The purpose of this phase was twofold: Familiarization of stimuli prior to labeling usually boosts levels of retention (e.g., Hilton and Westermann, 2016) and it lowers the task demand (e.g., Fennell, 2012).

After object familiarization, the child proceeded to the learning phase. During this ostensive-labeling phase, participants were taught a new word carrying either accent 1 or accent 2. The phase consisted of four trials of 30 s each. In the first and the third trial, the target appeared bouncing in front of a natural landscape and was labeled 10 times in each trial in sentences like: “Look! This is a [target]! A [target]! Can you see it? There’s the [target]!” In total, the child heard 20 repetitions of the target label. Presenting a number of repetitions is in line with previous research on retention of novel word-object mappings (e.g., Quam and Swingley, 2010; Singh et al., 2014; Hilton and Westermann, 2016). Note that the target label always appeared in focus-final position in a declarative sentence. In this way, the phonetic realization of the Limburgian tones was held constant, and the child thus did not have to abstract away from different surface realizations. In trials two and four, the distracter object appeared in the same scenario and was talked about for an equal amount of time, but crucially, it did not receive a label. We tried to encourage the child to wonder what the name of the distracter was. The target and distracter object were presented for an equal amount of time to prevent a familiarity preference for one of both objects in the subsequent test phase. The order of trials was the same across blocks and participants.

Following the learning phase, the child entered the test phase that consisted of four test trials and four filler trials. In test trials, the target and the distracter toy appeared side by side on the screen. Children were asked to “Look at the [target].” Target onset was always at 2500 ms to enable children to inspect both objects before naming and to establish a baseline preference. To maximize engagement, a second sentence like: “Can you find it?” followed 1000 ms after target offset. Test trials lasted 7 s.

In two of the test trials, the label for the target object was correctly pronounced [Correct Pronunciation (CP) trials], while in the other two, the label was mispronounced [Mispronunciation (MP) trials]. This MP involved a change in pitch: A word taught with accent 1 was mispronounced with accent 2 and vice versa. Recall that during test trials the novel target item was paired with a novel, unlabeled distracter item. The presence of a nameless distracter offered participants the possibility to consider the mispronounced version of the target label to be a novel label for the unlabeled distracter. This presupposes the use of the principle of mutual exclusivity (ME; Markman, 1990). This principle guides people to map novel words to unfamiliar rather than familiar referents. The use of ME to identify referents of novel words has been reliably demonstrated in infants from 16 months of age (e.g., Halberda, 2003) and in monolingual, bilingual, and bidialectal preschool children (e.g., Markman and Wachtel, 1988; Diesendruck and Markson, 2001; Durrant, 2014; Singh et al., 2014; Kalashnikova et al., 2015). The procedure with a novel target and a novel distracter object has been successfully applied in similar word learning studies with 1.5- to 2-year-olds (Singh et al., 2014), 2.5-year-olds (Quam and Swingley, 2010), and 3- to 5-year-olds (Singh and Quam, 2016).

Order of test trials was pseudo-randomized in such a way that the target would never appear on the same side more than twice in a row. Moreover, all children were presented at least one CP trial before the first MP trial. This resulted in three trial orders. To make sure children would remain engaged in the task, four filler trials involved correct pronunciations of four well-known words (e.g., Singh et al., 2015; Buckler and Fikkert, 2016). Test phases across all versions started with a filler trial to help children understand the nature of the task. Test and filler trials were presented in an alternating fashion.

Between blocks, children watched a 1-min video featuring farm animals and animal noises. The second block had the same structure as the first block but featured a new object-pair, one of which would receive a novel label. Object labels and tones were counterbalanced across participants. Each child was tested on his/her sensitivity to tonal MPs of accent 1 and accent 2 to test for asymmetries in tone sensitivity (e.g., Francis and Ciocca, 2003; Shi et al., 2017). Throughout the experiment, trials were preceded by a purple flashing light in the screen center and were initiated once the child fixated the attention getter.

#### Stimuli

For this experiment, we created two pseudo-word pairs: *taaf*_1/2_ [ta:f] and *moon*_1/2_ [mo:n].^7^ We decided to teach each participant two words instead of one to reduce the possibility that any effects were idiosyncratic to a particular word. Moreover, in this way all participants could learn one word with accent 1 and one word with accent 2.

The segments and phonotactics of the target stimuli were equally compatible with Limburgian and Dutch, and both pseudo-word pairs were derived from existing tonal minimal pairs in Limburgian to ensure that they were legal with both tones.^8^ Additionally, we controlled for phonological neighborhood density, since the existence of phonological neighbors could hinder children from using their full phonological sensitivity (e.g., Swingley et al., 1998; Swingley and Aslin, 2007) or from using the principle of ME (e.g., Jarvis et al., 2004). We considered a word a phonological neighbor if the item differed from the novel word by substituting, adding or deleting a single phoneme (Luce and Pisoni, 1998; Swingley and Aslin, 2002). We only considered words from the Lexilijst Nederlands (Schlichting and Spelberg, 2002) that are supposed to be produced and known by 15- to 27-month-old Dutch children. *Taaf* had no phonological neighbors known to children of this age, whereas *moon* had one phonological neighbor for the Dutch participants (*maan* [ma:n], ‘moon’), and two for the Limburgian participants (*maon*_1_ [mɔ:n], ‘moon’; *sjoon*_2_ [ʃo:n], ‘shoe’).

Carrier sentences were recorded in Limburgian and Dutch. Target stimuli were recorded in and spliced from Limburgian carrier sentences to guarantee tone accuracy.^9^ All stimuli were recorded in a child-friendly way by a female native speaker of Dutch and of an East-Limburgian dialect spoken in the municipality of Roermond. She reported to be dominant in Limburgian, but was equally proficient in Dutch and was trained in speaking accentless Standard Dutch. For Limburgian children, pre-experimental instructions as well as the experiment itself were in Limburgian. For Dutch children, the entire procedure was in Dutch. Across language contexts, only the tokens of the target stimuli *taaf* and *moon* were the same. Care was taken that the Dutch and Limburgian stimuli were recorded with the same intent and enthusiasm. The target stimuli were recorded multiple times with accent 1 as well as accent 2 and always appeared in a declarative focus-final context to avoid differences in the phonetic realization of the tones. Recordings were made in a sound-attenuated booth using Adobe Audition (version CS6, 44.1 kHz). Stimuli were equalized for intensity to 65 dB and prepared for the experiment using Praat (version 5.3.35; Boersma and Weenink, 2012). For stimuli excision we followed the guidelines presented in Turk et al. (2006).

In total, 12 tokens of *taaf*_1_, *taaf*_2_, *moon*_1,_ and *moon*_2_ were selected, based on intuition of a native speaker of an East-Limburgian dialect [the first author] and careful listening by a trained phonetician [Carlos Gussenhoven]. Ten tokens were used in the learning phase, the remaining two in the CP trials in the test phase. For all tokens we measured maximum and minimum f0, f0 range (max f0 to min f0), average f0, and duration of the tone bearing portion as well as the duration of the entire token. Measurements were done manually, taking auditory as well as spectral properties into account. Independent *t*-tests revealed that accent 1 and accent 2 tokens differed significantly from each other with respect to minimum f0, maximum f0, and f0 range (see **Table A3** in the **Appendix**).

The four filler trials involved correct pronunciations of known words. One filler pair consisted of a cow and a horse, and the other of a car and a ball. Items were chosen for their very high frequency in the productive vocabulary of the age group at test, according to the Lexilijst Nederlands (Schlichting and Spelberg, 2002).

**FIGURE 5:**
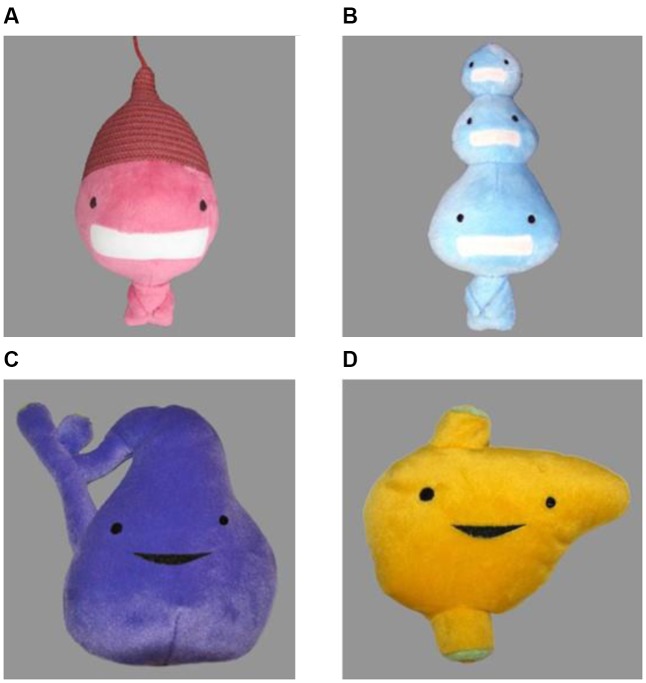
The visual target stimuli used in the experiment. Objects **(A,B)** always appeared as a pair as well as objects **(C,D)**.

The visual target stimuli consisted of four plush toy objects of an animate character (see **Figure 5**). All objects had different, vibrant colors (pink, blue, purple, and yellow) and shapes. The pink and blue object (**Figures 5A,B**) were paired as well as the purple and yellow object (**Figures 5C,D**). Pairs were matched in visual complexity, brightness, and size. A paired-samples *t*-test comparing the mean proportion of looking time toward the target (*M* = 0.51, *SD* = 0.08) and the distracter object (*M* = 0.50, *SD* = 0.08) during the object familiarization phase showed that participants did not show a preference for the target object prior to the learning (i.e., labeling) phase [*t*(57) = 0.59, *p >* 0.05].

In the object familiarization phase and the test phase, the stimuli consisted of photographs of the objects against a gray background. During the learning phase, the objects bumped up and down against the background of a natural scene. Filler stimuli in the test phase consisted of photographs of a horse, a cow, a car, and a ball against a gray background. Two different pictures per object were used across blocks to minimize boredom effects.

#### Data Pre-processing and Analysis

Children’s video recordings were coded offline using ELAN (version 4.5.0; Wittenburg et al., 2006) with a resolution of 40 fps. In test trials, target onset was always at 2500 ms. The 2500 ms window prior to target onset was labeled the pre-naming window. The post-naming window lasted 2000 ms, starting 367 ms after target onset (e.g., Swingley and Aslin, 2000; Quam and Swingley, 2010; Altvater-Mackensen et al., 2013; Singh et al., 2014). The coder was blind to trial type and target side. A random 20% of the videos was recoded by a second experienced coder. The correlation between two coders was very strong (Pearson’s *r* = 0.801, *p* < 0.001).

To ensure that our analyses were based on clean data and to enable within-subject comparisons of CP vs. MP trials and of accent 1 vs. accent 2 words, we maintained a number of trial, block, and participant exclusion criteria. **Table A1** in the **Appendix** provides a detailed overview of exclusion.

Test trials were excluded if (1) a child looked less than 500 ms during the 2000 ms post-naming window (e.g., Quam and Swingley, 2010; Singh et al., 2014; Tsuji et al., 2016), (2) the participant fixated only one of two objects during the 2500 ms pre-naming window (e.g., White and Morgan, 2008; Mani and Plunkett, 2011; Singh et al., 2015; Buckler and Fikkert, 2016), (3) an equipment or experimenter error occurred, and (4) if a participant refused to participate (e.g., by getting up and walking around) and the experiment had to be aborted.

A block was excluded if (1) a participant did not contribute at least one valid trial per condition (CP and MP) during the test phase (e.g., Buckler and Fikkert, 2016; Tsuji et al., 2016), and (2) total looking time during target and/or distracter learning trials was under 20 s out of a total of 60 s (e.g., Tsuji et al., 2016). The latter criterion is based on the assumption that children who pay more attention to the novel objects during learning should be better able to retain the novel word-object mapping (Hilton and Westermann, 2016).

Participants were excluded from the analyses if (1) at least one block had to be excluded, (2) an equipment failure or experimenter error occurred, and (3) other conditions were not met, e.g., if a participant’s linguistic background was inappropriate or if we had twin participants.

Children’s target recognition was inferred from the presence of a naming effect that is typically measured as an increase in target fixation upon hearing the target label relative to a baseline looking measure (e.g., Swingley and Aslin, 2000; Singh et al., 2015). To calculate the naming effect, the increase in the proportion of target looking (PTL) between the pre-naming and post-naming window of a test trial was calculated [i.e., Post-naming_PTL(T/[T+D])_ – Pre-naming_PTL(T/[T+D])_], resulting in a difference score. Computing naming effects by taking each individual participants’ pre-naming values into account serves to control for possible effects of preference for a particular stimulus (e.g., White and Morgan, 2008; Quam and Swingley, 2010; Mani and Plunkett, 2011; Singh et al., 2015). A paired-samples *t*-test showed a small yet significant difference in PTL between object familiarization phase (*M* = 0.51, *SD* = 0.08) and pre-naming window (*M* = 0.53, *SD* = 0.07), *t*(57) = -2.05, *p* = 0.045, Cohen’s *d* = -0.27. Moreover, a one-sample *t*-test showed that pre-naming PTL differed significantly from chance: *t*(57) = 3.56, *p* = 0.001, Cohen’s *d* = 0.47. Thus, it appears that the target object had become slightly more interesting than the distracter after the learning phase due to repeated labeling (e.g., Schafer and Plunkett, 1998). To control for a possible effect of this target preference, we chose the post-minus pre-naming PTL measure as our dependent variable.

Naming effects were calculated and compared for CP and MP trials. If children notice the MP, the naming effect will be significantly less strong in MP than in CP trials. However, it is important to inspect the naming effect in MP trials more closely to gain insight into the strength of the MP effect. First, even if the naming effect in MP trials is significantly weaker than the naming effect in CP trials, it can still be positive and significantly above zero (as attested for one-feature segmental MPs in White and Morgan, 2008). This indicates that target recognition is hindered to some extent, but that recognition still takes place. Secondly, the naming effect in MP trials might not differ significantly from 0, signaling uncertainty, meaning that target recognition is hindered to such extent that recognition fails (as attested for two- and three-feature segmental MPs in White and Morgan, 2008, and for tonal MPs in Singh et al., 2014, 2015). Thirdly, a significant negative naming effect would point to a preference for the distracter object and can be seen as evidence for the formation of a novel mapping between the auditory label and the distracter object based on ME (e.g., Swingley and Aslin, 2000; White and Morgan, 2008; Mani and Plunkett, 2011).

## Results

**FIGURE 6:**
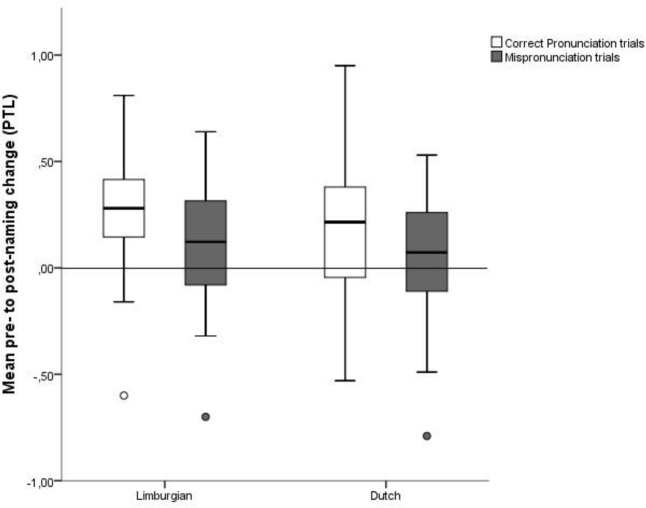
Mean pre- to post-naming change (PTL) in CP and MP trials for Limburgian and Dutch toddlers.

**Figure 6** shows naming effects for Limburgian and Dutch toddlers in the CP and MP condition.

To ensure whether word learning was successful, the naming effect in CP trials was compared to zero for each group by means of a one-sample *t*-test. For both Limburgian and Dutch toddlers, there was a significant positive naming effect in CP trials (Limburgian: *M* = 0.25, *SD* = 0.15, *t*(22) = 8.28, *p* < 0.001, Cohen’s *d* = 1.73; Dutch: *M* = 0.18, *SD* = 0.23, *t*(34) = 4.60, *p* < 0.001, Cohen’s *d* = 0.78). From this we can conclude that both participant groups learned the novel word-object mapping.

Next, a three-way mixed ANOVA with Condition (CP vs. MP) and Tone (Accent 1 vs. Accent 2) as within-subjects factors and Language (Limburgian vs. Dutch) as the between-subjects factor was conducted to evaluate the possible influence of language and pitch change on the naming effect. Results revealed a significant main effect of Condition, *F*(1,56) = 8.53, *p* = 0.005, $\eta_{p}^{2}$ = 0.13, observed power = 0.82, with a significantly stronger naming effect in CP trials (*M* = 0.21, *SD* = 0.20) than in MP trials (*M* = 0.09, *SD* = 0.24). No other significant main effects or interactions were found (all *p*s > 0.1). Both Limburgian and Dutch children thus treated the pitch change as lexically relevant as indicated by a significantly weaker naming effect in MP trials compared to CP trials. Mean PTL values and standard deviations for pre- and post-naming windows per Condition and Language are listed in **Table 1**.

**Table 1:** Mean proportion of target looking in pre- and post-naming windows per group and condition for the toddlers.

PTL (SD)	Limburgian	Dutch
CP Pre-naming	0.51 (0.08)	0.51 (0.10)
CP Post-naming	0.76 (0.13)	0.69 (0.20)
MP Pre-naming	0.58 (0.10)	0.55 (0.10)
MP Post-naming	0.70 (0.20)	0.62 (0.22)

*Standard deviations in parentheses*.

To investigate the strength of the MP, the naming effect in MP trials was compared to zero by means of a one-sample *t*-test. The test revealed a significant positive naming effect (*M* = 0.09*, SD* = 0.24; *t*(57) = 2.81, *p* < 0.01, Cohen’s *d* = 0.37). Thus, despite the naming effect being weaker in MP than CP trials, target recognition was still possible in MP trials. From this we can infer that the pitch change only hindered word recognition to a minor extent.^10^

We next tested Limburgian and Dutch adults in the same experiment to find out whether the sensitivity to pitch in both the Limburgian and Dutch children in Experiment 1 was adult-like or whether it reflected a not yet fully developed phonological system.

## Experiment 2

As with the Limburgian children, we expected Limburgian adults to notice a change in tone, but it was an open question how strongly it would hinder word recognition. Adult speakers might have learned not to rely on pitch too much during online language comprehension because of the relatively low functional load of lexical tone and because pitch has no lexical relevance in their second L1, Dutch.

Speakers of Dutch were expected not to attend to pitch during the recognition of newly learned words. However, if the sensitivity exhibited by the Dutch children was dependent on their knowledge of pitch as a cue to word stress and/or intonation, Dutch adults might still be sensitive to pitch differences by virtue of their accumulated experience with the native prosodic system (but see Quam and Swingley, 2010).

### Materials and Methods

#### Participants

Limburgian adults were recruited and tested in a public library in Roermond. The Limburgian listeners (*N* = 14, 5 males) ranged in age from 26 to 72 years (*M* = 53.6 years). An additional 10 participants were excluded from the analysis because (1) they reported to speak a dialect other than one from the East-Limburgian dialect region (*N* = 4), (2) they could only contribute one of two blocks due to exclusion of test trials (*N* = 3), or (3) they failed to learn the novel word-object mapping in one or two blocks, signaled by a mean PTL equal or smaller than 0.50 in the post-naming window of CP trials (*N* = 3).^11^ All included Limburgian participants were born and raised in the East-Limburgian dialect region and lived there at the time of test. All of them reported to actively use an East-Limburgian dialect. The Limburgian participants also had native command of Dutch, except for two participants who reported very good or good command. All of them can thus be considered bidialectals.

Dutch adults were recruited at Radboud University, Nijmegen, Netherlands, and tested at the Baby Research Center of the same university. The Dutch listeners (*N* = 22, 7 males) ranged in age from 18 to 40 years (*M* = 23). None of them had weekly contact with people speaking a Limburgian dialect in their presence. Moreover, none of them grew up or lived in the province of Limburg. An additional two participants were excluded from the analysis due to the exclusion of one of both blocks.

All Limburgian and Dutch participants reported some degree of non-native command of one or more non-tonal languages (i.e., English, German, French, Spanish, Arabic, and Polish) as indicated on a six-point scale ranging from poor to native command, but none of them had experience with a tone language. All participants reported normal hearing and no speech, language, or attention deficits. Because of the fact that musical experience can have an influence on pitch processing (e.g., Burnham and Brooker, 2002; Burnham et al., 2015), we kept the number of musically trained individuals comparable across groups. Six of the Limburgian participants (43%) and eight of the Dutch participants (36%) reported to have had over 3 years of musical training. Ethical approval for the study was obtained from the Ethics Assessment Committee (EAC) of the Faculty of Arts at Radboud University, Nijmegen, Netherlands. Participants signed an informed consent and took part in the experiment either voluntarily or for a small fee.

#### Apparatus, Stimuli, and Procedure

The apparatus, stimuli, and procedure of the adult experiment were comparable to Experiment 1, as in Quam and Swingley (2010), who also tested children and adults under similar conditions. For the Limburgian adults we used the same portable set-up as the Limburgian children, but they were tested in a quiet, darkened room in a public library. To minimize external interference, stimuli were presented through noise-canceling headphones (Sennheiser HME 110). Dutch adults were tested under the exact same conditions as the Dutch children.

Regarding the procedure, we added extra filler trials (16 instead of 4) to the test phase to distract adult participants’ attention away from the purpose of the experiment, leading to a total number of 20 trials. Participants were told before the study that they would be helping to test an experiment designed for 3-year-olds.

A paired-samples *t*-test, comparing the mean PTL toward the target (*M* = 0.51, *SD* = 0.05) and the distracter object (*M* = 0.49, *SD* = 0.05) during the object familiarization phase, showed that adult participants did not show a preference for the target object prior to the learning phase [*t*(35) = 0.73, *p* > 0.1]. After the experiment, adults completed a language background questionnaire.

#### Data Pre-processing and Analysis

A random 20% of the adult videos was recoded by a second experienced coder. Inter-coder reliability was excellent (Pearson’s *r* = 0.937, *p* < 0.001).

Post-naming PTL was calculated within a 1000 ms window, starting 367 ms after target onset. We could have shifted the analysis window for adults earlier in time, but since earlier studies have shown that this does not have consequences for the results (e.g., Swingley, 2009), we retained the starting point of 367 ms post-target onset.^12^

As with the child data, we found a significant difference in PTL during object familiarization (*M* = 0.51, *SD* = 0.05) and pre-naming window (*M* = 0.56, *SD* = 0.12), *t*(35) = -2.73, *p* = 0.01, Cohen’s *d* = -0.45. Moreover, a one-sample *t*-test showed that pre-naming PTL differed significantly from chance: *t*(35) = 3.16, *p* = 0.003, Cohen’s *d* = 0.53. Thus, it appears that also for the adults the target object had become more interesting than the distracter after the learning phase. We again chose the post-naming minus pre-naming PTL measure as our dependent variable.

## Results

**FIGURE 7:**
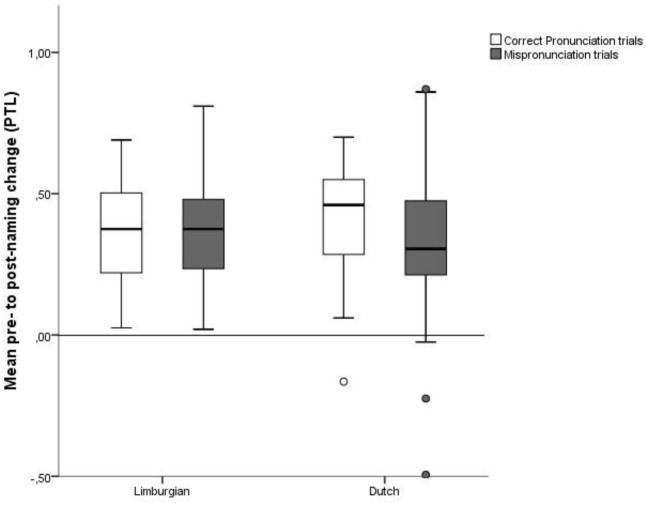
Mean pre- to post-naming change (PTL) in CP and MP trials for Limburgian and Dutch adults.

Naming effects for Limburgian and Dutch adults in CP and MP conditions are depicted in **Figure 7**.

To ensure that the adult participants successfully learned the novel word-object pairings, the naming effect in CP trials was first compared to zero for each language group by means of a one-sample *t*-test. For both Limburgian and Dutch adults, there was a significant positive naming effect in CP trials [Limburgian: *M* = 0.36, *SD* = 0.13, *t*(14) = 10.69, *p* < 0.001, Cohen’s *d* = 2.86; Dutch: *M* = 0.41, *SD* = 0.14, *t*(22) = 14.28, *p* < 0.001, Cohen’s *d* = 3.04]. From this we can conclude that both participant groups learned the novel word-object mappings.

Next, a three-way mixed ANOVA with Condition (CP vs. MP) and Tone (Accent 1 vs. Accent 2) as within-subjects factors and Language (Limburgian vs. Dutch) as the between-subjects factor was conducted. The analysis yielded no main effects or interactions (all *p*s > 0.05).

As in the CP trials, the naming effect in MP trials was significantly above zero [*M* = 0.34, *SD* = 0.22; *t*(38) = 9.53, *p* < 0.001, Cohen’s *d* = 1.53].

The absence of an effect of Condition or Language is probably due to participants showing very strong naming effects in both CP and MP trials, as becomes clear from the PTL measures in **Table 2**. As can be inferred from Quam and Swingley (2010), the procedure used should be sensitive enough to yield a vowel MP effect. However, Quam and Swingley (2010) did not test native tone language speakers and thus did not show whether the method is equally suited to yield sensitivity to a change in pitch. This means that we cannot rule out the possibility that our findings are due to a task effect.

**Table 2:** Mean proportion of target looking in pre- and post-naming windows per group and condition for the adult participants.

PTL (SD)	Limburgian	Dutch
CP Pre-naming	0.56 (0.12)	0.52 (0.13)
CP Post-naming	0.93 (0.12)	0.94 (0.10)
MP Pre-naming	0.59 (0.17)	0.58 (0.14)
MP Post-naming	0.94 (0.10)	0.93 (0.14 s)

*Standard deviations in parentheses*.

Our adult data thus provide no evidence of an effect of pitch variation on the recognition of newly learned words.

## Discussion

In this study, we asked whether pitch plays a larger role in novel word learning and recognition in children acquiring East-Limburgian compared to a control group of children acquiring Standard Dutch. To see whether their interpretation of pitch was adult-like or not yet fully developed, we also tested Limburgian and Dutch adults.

Our main finding is that both Limburgian and Dutch children pay attention to pitch changes in newly learned words. However, children still preferred the target over the distracter object upon hearing a pitch change, indicating that a change in tone did not hinder word recognition to a great extent. Regarding our adult data, we can conclude that both Limburgian and Dutch adults succeeded in learning novel word-object mappings. However, we cannot draw conclusions about their interpretation of pitch changes due to very strong naming effects in both CP and MP conditions. In the next section, we will first discuss the findings from Experiment 1 with Limburgian and Dutch toddlers.

### The Lexical Encoding of Pitch in Limburgian and Dutch Toddlers

The finding that Limburgian children were sensitive to MPs involving pitch was in line with previous word recognition studies with tone language learners (Singh et al., 2014, 2015). However, as signaled by the positive naming effect in MP trials, the pitch change did not inhibit target recognition. This pattern of results is in line with toddlers’ responses to one-feature segmental MPs in White and Morgan (2008). However, previous studies investigating Mandarin found no naming effects in tonal MP conditions (Singh et al., 2014, 2015), suggesting that pitch changes are more detrimental to word recognition in Mandarin than in Limburgian. We would like to suggest three explanations for this finding.

First, the fact that Limburgian children recognized the target word despite a tonal change might be due to the relatively low functional load of tone. One of the factors contributing to the functional load of a contrast is the number of minimal pairs. The low frequency of tonal minimal pairs, plus the fact that listeners can mostly rely on sentence context for disambiguation, might mitigate the reliance on pitch in perceiving Limburgian. Similar explanations have been put forward by Cutler (1986) for the role of lexical stress in English and by Cutler and Otake (1999), Sekiguchi and Nakajima (1999), and Goss (2015) for the influence of pitch-accent on word recognition in Japanese. This reasoning is in line with the hypothesis that phonological category learning is driven by contrast in the vocabulary (Dietrich et al., 2007). However, Dietrich et al. (2007) argue on the basis of the results of a word recognition study that 18-month-olds’ native-like performance cannot have been the result of top-down information from the lexicon. The tested age group did not seem to know many minimal pairs involving the distinctions at test. We thus cannot assume that children need minimal pairs to decide whether a contrast is phonologically meaningful or not.

A second explanation for the Limburgians’ lenient treatment of MPs might be tonal surface variability. Recall that Limburgian listeners are confronted with a considerable amount of *allotonic* variation in lexical tone contours, but this variation cannot be ignored since it does signal meaningful information at the post-lexical level. In light of this pitch variation, it could be a challenge to recover the underlying tone system, at least for young learners (Demuth, 1995; Ota, 2003; Rost and McMurray, 2010). A replication of our study with Swedish children could provide additional insight into the effect of surface variation on developing tonal representations.

A third factor that may have influenced our Limburgian participants’ behavior is variation due to their exposure to multiple (closely related) linguistic varieties. Hardly any studies on the mapping of sounds to meaning focused on children acquiring two languages, let alone on children acquiring multiple dialects or regional varieties of the same language (for a review, see Fennell et al., 2016). One type of variation due to bidialectalism comes from exposure to different dialects and Limburgian-accented Dutch. Evidence for the effects of dialect-related variation on the phonological representation of known words is scarce. Durrant et al. (2015) showed that variable phonological input as a result of dialect variation has an impact on the specificity of lexical representations in 20-month-old British English multidialectal toddlers. In a preferential looking paradigm, they were tested on their sensitivity to single feature MPs of monosyllabic known words. MPs involved changes of onset consonants or of the vowel nuclei that were phonemic in all the varieties at test. The authors’ main finding was that multidialectal infants, other than monodialectal infants, did not treat MPs of familiar words differently from CP’s, suggesting that long-term exposure to regional linguistic variation leads to a broadening of phonetic categories or poorer use of phonological information in word recognition.

Another type of variation due to bidialectalism stems from lexical overlap. Limburgians know many cognates that do not have a tonal specification in Dutch. As such, they receive mixed evidence for the lexical relevance of pitch. Possibly, this mixed evidence (temporarily) leads them to assign less weight to pitch as a lexically contrastive feature. The existing evidence points in another direction, though. Van der Feest and Johnson (2016) tested 24-month-old Dutch toddlers who received mixed distributional evidence for the lexical contrastivity of fricative voicing. Toddlers were exposed to Limburgian-accented Dutch (which maintains the fricative voicing contrast) and to Dutch as spoken in the Nijmegen region (where the fricative voicing contrast is neutralized). Children treated fricative voicing as lexically relevant only in a Limburgian-accented context. The authors conclude that toddlers who receive mixed evidence for a phonological contrast due to variation in accents in their input do not simply treat the contrast as allophonic, nor do they ignore the contrast. Rather, they appear to track two sets of statistics, one for each variant, as bilingual children have been argued to do (e.g., Sundara and Scutellaro, 2011). Studies showing that the presence of mixed distributional evidence for a lexical tone contrast does not lead to less specific lexical representations were carried out by Singh et al. (2014, 2016). Twelve- to thirteen-month-old Mandarin-English bilinguals who, like our Limburgian participants, received mixed evidence for the lexical relevance of pitch, noticed tonal MPs in a Mandarin version of the one-object switch-task, but not in a non-tonal English version (Singh et al., 2016). In a preferential looking paradigm, also 18- and 24-month-old Mandarin-English bilinguals were sensitive to tonal MPs (Singh et al., 2014; but see Singh and Quam, 2016, for different results in a task involving language switching). From these findings we can probably infer that our Limburgian participants’ lenient treatment of tonal MPs was not the result of their exposure to non-tonal cognates in Dutch. It could, however, be the case that their long-term exposure to dialect-related variation leads to a more general relaxation of phonetic boundaries, leading to less well specified lexical representations (e.g., Durrant et al., 2015). To investigate if the latter explanation holds, future studies should test Limburgians’ responses to a variety of tonal and segmental MPs of familiar words, similar to the Durrant et al. study.

The fact that Dutch toddlers responded to pitch variation in a word learning task is not in line with previous studies on the lexical encoding of tone in non-tone language children (e.g., Quam and Swingley, 2010; Singh et al., 2014; Hay et al., 2015). These studies have shown that, from some point in development, English toddlers ignore pitch information during word learning. However, comparisons to these prior studies are difficult because these studies did not directly compare performance of tone and non-tone language learning children (i.e., in one statistical analysis). Moreover, prior studies testing non-tone language children have been restricted to learners of English, making it impossible to generalize their results to all non-tone language learners. We want to put forward three explanations for Dutch toddlers’ sensitivity to word-level pitch.

First, Dutch toddlers could have interpreted the Limburgian pitch patterns as post-lexical intonation, as has also been put forward as an explanation for successful lexical tone discrimination in Ramachers et al. (2017). More specifically, toddlers might over-assign weight to post-lexical factors in novel word learning tasks by virtue of having observed their communicative significance at other levels of linguistic structure (e.g., Singh et al., 2014; Hay et al., 2015). Similarly, Braun et al. (2014) proposed that extensive utterance-level prosody in the L1 is helpful for storing pitch information as part of novel mental representations. On the other hand, Frota et al. (2012) showed that, by age 3, European Portuguese children do notice stress changes, but no longer treat intonation changes in newly learned words as lexically relevant.

A second possible explanation for the behavior of the Dutch toddlers also relates to L1 intonation. In a word recognition study, Fikkert and Chen (2011) showed that Dutch 24-month-olds have knowledge of appropriate native intonation patterns. Particularly in imperatives, Dutch toddlers strongly preferred a high-low pitch pattern combined with a strong-weak (trochaic) stress pattern. In our study, the target sentences in the test trials were always imperatives. Possibly, our Dutch toddlers’ behavior could have been influenced by their expectations of what a well-formed imperative sounds like. An imperative that ends in a high-low pitch pattern (i.e., accent 1) could be preferred over an imperative ending in a low-high pitch pattern (i.e., accent 2). This would result in Dutch children structurally fixating the target *less* if pronounced with accent 2, *regardless* of the trained tone. In this case we should have found an interaction involving our variables Language and Tone. Since we attested no such interaction, our data provide no evidence for the suggestion that Dutch children’s expectations regarding well-formed imperatives have influenced their behavior in our study.

The third explanation of the fact that Dutch toddlers noticed a pitch change in a novel word is that they might have perceived the Limburgian tone contrast as a quantity contrast rather than as a pitch contrast. Previous research has shown that the shape of a pitch pattern can indeed affect the perceived duration of the tone bearing vowel (e.g., Lehiste, 1976; Pisoni, 1976; Yu, 2010; Gussenhoven and Zhou, 2013). Despite the fact that the Limburgian tones’ primary acoustic cue is pitch rather than duration, we think it is possible that speakers of Dutch perceived the pitch difference as a difference in duration. Previous research has shown that native and non-native speakers may give different degrees of attention to acoustic cues under the influence of the different functions and/or distributions of these cues in the L1 (Gandour and Harshman, 1978; Cebrian, 2006; Ueyama, 2000). For example, Gandour and Harshman (1978) showed cross-linguistic differences in the importance attributed to duration as a cue for tone perception, presumably reflecting the different linguistic status of vowel duration in their participants’ L1s. In light of the fact that duration is an acoustic cue to lexical contrast in Dutch (i.e., word stress and vowel quantity) and Dutch children’s early sensitivity to these contrasts (e.g., Dietrich et al., 2007; de Bree et al., 2008), we propose that the Dutch children in our study could have drawn upon their knowledge of this cue when perceiving a non-native tone contrast.

Anecdotal evidence with adult speakers of Dutch seems to strengthen this claim. Naïve speakers of Dutch who imitate the Limburgian tones tend to lengthen the stressed syllable of accent 2 words relative to accent 1 words (e.g., Ueyama, 2000). The impression that the citation form of accent 2 is longer in duration than the respective accent 1 form could be due to the more complex pitch pattern of accent 2 (H^∗^LH) compared to accent 1 (H^∗^L), assuming that changes in f0 can go hand in hand with a perceptual increase in duration (e.g., Lehiste, 1976; Rietveld and Gussenhoven, 1987; but see Gussenhoven and Zhou, 2013). In fact, Heijmans (2003) reports a formerly tonal dialect just outside the East-Limburgian area in which the tonal contrast was in large part reinterpreted as a length contrast. In future research, Dutch listeners could be presented tonal minimal pairs and explicitly judge which one sounds longer (e.g., Lehiste, 1976).

Until now, we have assumed different explanations for the behavior of the Limburgian and Dutch toddlers, despite their behavior being comparable. Lastly, we would like to mention the possibility that their behavior can be based on the same explanation. Recall that the only prosodic difference between Limburgian and Dutch is the fact that pitch is lexically relevant in Limburgian. Both languages make use of vowel duration, word stress, and intonation. We therefore cannot exclude the possibility that the Limburgians might not perceive the difference between accent 1 and accent 2 as a pitch contrast, but as a durational contrast.

Another finding that deserves some attention, especially in light of ongoing typological discussions about the phonological status of the Limburgian word prosodic contrast (e.g., Köhnlein, 2016, and references therein), is that Limburgian children were sensitive to MPs of both accent 1 and accent 2. Gussenhoven and Peters (2008) assume that accent 2 is the lexically specified tone, but our data provide no evidence for a perceptual asymmetry due to lexical (under)specification of one of the accents. It is possible that we did not attest an asymmetry due to a lack of power. However, an inspection of the means did not reveal a trend toward such an asymmetry. More research is needed to draw conclusions on this matter.

In the next section, we will turn to the findings from Experiment 2 with Limburgian and Dutch adults.

### The Lexical Encoding of Pitch in Limburgian and Dutch Adults

In line with Quam and Swingley (2010), who used a very similar design, the Limburgian and Dutch adults in our study successfully learned novel word-object pairings. However, both groups showed very strong naming effects in both CP and MP trials, possibly masking effects of Condition and/or Language. Their high recognition scores could either mean that the task was not sensitive enough [but see Quam and Swingley (2010)], or that our participants did not notice a pitch change within a word, or both.^13^

Besides the pitch change condition, Quam and Swingley (2010) also included a vowel MP condition. In this condition, English participants exhibited a marginally significant negative naming effect, whereas they showed a significant positive naming effect in both the pitch MP and in the CP condition. Their effect of Condition thus rested on the significant negative naming effect induced by the vowel MP. They found no significant difference between the performance in pitch MP and CP conditions, which is in line with the behavior of our participants. In a future study, it would be valuable to include one or more segmental MP conditions in addition to a tonal MP condition (e.g., Quam and Swingley, 2010; Singh et al., 2014, 2015).

With respect to our Limburgian participants, it could be that lexical tone in Limburgian, relative to segments, does not share equal priority as a cue to word recognition. A similar claim has been made for Japanese (e.g., Goss, 2015). Since adult Limburgians have accumulated ample linguistic experience, they might have learned not to rely heavily on pitch during online language comprehension because of the relatively low functional load of lexical pitch and/or because pitch has no lexical relevance in their second L1, Dutch. However, in light of Braun et al.’s (2014) finding, who showed that adult speakers of German were very sensitive to Mandarin tone contrasts in a word learning paradigm, we strongly believe that the absence of effects in our study is due to task effects. To increase the demands on memory load in a future task, we could use disyllabic stimuli and/or teach participants multiple tonal minimal pairs simultaneously (e.g., Braun et al., 2014).

Due to the lack of effects of Language, Condition or Tone in the adult study, we cannot draw conclusions on the phonological status of the Limburgian tone contrast. A lexical accent correctness judgment task (e.g., Goss and Tamaoka, 2015) or a lexical decision task with either phonological priming (e.g., Cutler and Otake, 1999) or semantic priming with tonal MPs could potentially advance our understanding of the lexical status of the Limburgian word prosodic contrast.

One important limitation that we want to mention at this point pertains to the input that both child and adult Limburgian participants were exposed to during the learning phase of the current experiment. Recall that they were presented with multiple tokens of the target word, but that the prosodic context was held constant. That is, participants did not have to deal with surface variation with which they are usually confronted due to tone-intonation interactions in natural language input. It would be interesting to see how Limburgian toddlers and adults would perform if this surface variation were included in the learning phase.

## Conclusion

Both Limburgian and Dutch 2.5- to 4-year-old children are sensitive to lexical pitch information in novel words. This indicates that they store pitch information as part of their novel lexical entries. Due to a lack of effects in our adult study, we cannot draw conclusions on the lexical encoding of pitch in Limburgian and Dutch adults. Since pitch is not contrastive at the word-level in Dutch, Dutch listeners should recognize words irrespective of their pitch pattern. Dutch toddlers’ sensitivity to word-level pitch probably reflects their growing knowledge of the native prosodic system. They could either have perceived the different pitch patterns in terms of intonation (e.g., Singh et al., 2014), or in terms of vowel duration. The Limburgian toddlers’ behavior was in line with our expectations since pitch is assumed to be part of Limburgian lexical representations. The fact that a pitch change only hindered word recognition to a minor extent, and possibly not at all in Limburgian adults, could be due to the specific input conditions that Limburgians are exposed to. Future studies could include speakers of Swedish, since word-level pitch in Swedish also has a relatively low functional load and also shows a relatively high amount of surface variation, to corroborate that functional load and phonetic variability indeed have an impact on lexical tone processing.

## Author Contributions

SR conceptualized the study, recruited participants, collected data, conducted data analyses, interpreted the results, and drafted the manuscript. SB conducted data analyses, interpreted results, and revised the manuscript. PF assisted in the conceptualization of the study, interpreted results, and revised the manuscript.

## Conflict of Interest Statement

The authors declare that the research was conducted in the absence of any commercial or financial relationships that could be construed as a potential conflict of interest.

## Footnotes

1 Note that *Limburgian* is an umbrella term for many different dialects. Not all of these dialects have lexical tone, which will be discussed in detail later on.

2 From personal communication with the authors, we know that the second language of the Mandarin bilinguals was English.

3 www.cbs.nl

4 Note that some scholars have questioned whether the Limburgian accents come from lexical tones. They argue that there is no lexical tone in Limburgian, but that the contrast emerges from different foot structures (e.g., Köhnlein, 2016).

5 Henceforth, the term *Limburgian* is used to refer to those Limburgian dialects that use lexical tone.

6 This questionnaire is a translation/adaptation of the Questionnaire for Parents of Bilingual Children (COST Action IS0804, 2011). It is the short version of a longer questionnaire piloted by research groups in several countries within COST Action IS0804, which was in part based on the ALEQ (Paradis, 2011) and the ALDeQ (Paradis et al., 2010).

7 Subscripts indicate accents 1 and 2.

8 The pseudoword *taag* comes from Limburgian *graaf* [ddd eeea:f], meaning ‘grave’ with accent 2 and ‘count’ with accent 1. The pseudoword *moon* comes from Limburgian *sjoon* [ʃo:n], meaning ‘shoe’ with accent 2 and ‘beautiful’ with accent 1.

9 Note that some of the Limburgian stimuli were spliced too, since the selected tokens of the target stimuli did not always appear in the desired carrier sentence in the original recordings.

10 Some previous studies found age-related differences in the sensitivity to pitch changes in tone language learning bilinguals (e.g., Singh et al., 2015). Since we also tested tone language learning ‘bilinguals’ spanning exactly this age range, we ran an additional mixed ANOVA on our Limburgian sample including Age as a within-subjects variable, comparing younger (31–38 months, *N* = 11) to older (42–49 months, *N* = 12) children. The analysis yielded a main effect of Condition, *F*(1,21) = 4.63, *p* = 0.04 and a marginally significant Condition × Tone × Age interaction, *F*(1,21) = 3.21, *p* = 0.088, suggesting that the effect of Condition in the younger children is carried by the accent 2 items whereas in older children it is carried by the accent 1 items. No other significant main effects or interactions were attested (all *p*s > 0.05). As suggested by the reviewers, we also ran an ANCOVA including Age as a covariate. This analysis only yielded a marginally significant Condition × Tone × Age interaction, *F*(1,21) = 3.38, *p* = 0.08, suggesting that the effect of Condition only holds for accent 1 items. This could after all signal a trend toward a perceptual asymmetry, indicating that accent 1 is the lexically specified tone. Increasing the sample size could perhaps increase the significance of this result, but was outside the scope of our study.

11 The drop-out rate might be due to the testing conditions: Participants were personally invited to participate and had to interrupt what they were doing. Moreover, in contrast to typically tested student populations, our participants might not have known what to expect. Some of them might not have been that motivated but accepted the invitation to avoid disappointment. These factors could have influenced their attention during the experiment.

12 A *post hoc* inspection of the adults’ looking behavior in an earlier time window indeed showed that they were on target immediately after target onset. Changing the analysis window would thus not have changed the results.

13 Yet another factor that could have influenced the results are the specific testing conditions (see footnote 11). Possibly, participants were not attentive enough due to a lack of motivation.

## Appendix

**Table A1:** Number and percentage of excluded trials, blocks, and participants per language and age group for the child study.

	Dutch	Limburgian
**Trial exclusion n (%)**		
(1) <500ms LT post-naming	12 (7.5)	35 (21.3)
(20 PTL pre-naming = 1	22 (13.8)	23 (14.0)
(3) equip./exp. error	1 (0.6)	2 (1.2)
(4) Refusal to participate	4 (2.5)	14 (8.5)
Total excluded n (%)	38 (23.8)	74 (45.1)
*Total n trials*	160	164
**Block exclusion n (%)**		
(1) Not enough test trials	4 (5)	15 (18.3)
(2) <20 s LT learning	–	4 (4.9)
Total excluded n (%)	4 (5)	19 (23.2)
*Total n blocks*	*80*	*82*
**Participant exclusion n (%)**		
(1) 1 or 2 blocks excluded	4 (10)	18 (43.9)
(2) Equip./exp. error	–	–
(3) Other	1 (2.5)	–
Total excluded n (%)	5 (12.5)	18 (43.9)
*Total n participants*	*40*	*41*

**Table A2:** Means, standard deviations, and ranges of proportions of input quantity and quality for the Limburgian children (missing *N* = 1).

PaBiQ measures	Mean (SD)	Range
Input quantity Limburgian	0.70 (0.24)	0.15–1
Input quantity Dutch	0.40 (0.22)	0.02–0.72
Input quality Limburgian	0.49 (0.11)	0.31–0.69
Input quality Dutch	0.39 (0.13)	0.19–0.69

**Table A3:** Acoustic measurements of the target stimuli.

Measures	Accent 1 (*n* = 24)	Accent 2 (*n* = 24)	*p*-value
Min F0 TBP^∗^ (Hz)	168.5 (8.1)	209.3 (22.4)	<0.001
Max F0 TBP (Hz)	402.7 (32.1)	380.5 (20.0)	0.007
Mean F0 TBP (Hz)	294.4 (38.2)	296.5 (22.7)	>0.1
F0 range TBP (Hz)	234.2 (32.4)	171.2 (17.0)	<0.001
Duration TBP (s)	0.38 (0.08)	0.37 (0.06)	>0.1
Duration token (s)	0.55 (0.03)	0.57 (0.04)	>0.05

^∗^*TBP stands for tone bearing portion. Note that the TBP for *moon* stimuli consisted of the entire rhyme (i.e., [o:n]) whereas for *taaf* stimuli it consisted of the nucleus (i.e., [a:]). Standard deviations in parentheses*.
